# Liver abscess caused by *Clostridium perfringens* after left hepatic trisectionectomy for perihilar cholangiocarcinoma: a case report

**DOI:** 10.1186/s40792-023-01687-8

**Published:** 2023-06-19

**Authors:** Yuuko Tohmatsu, Mihoko Yamada, Shimpei Otsuka, Katsuhisa Ohgi, Ryo Ashida, Hanako Kurai, Haruna Yasui, Takashi Sugino, Katsuhiko Uesaka, Teiichi Sugiura

**Affiliations:** 1 Division of Hepato-Biliary-Pancreatic Surgery, Shizuoka Cancer Center, 1007 Shimo-Nagakubo, Nagaizumi-Cho, Sunto-Gun, Shizuoka 411-8777 Japan; 2 Division of Infectious Diseases, Shizuoka Cancer Center, Shizuoka, Japan; 3 Division of Pathology, Shizuoka Cancer Center, Shizuoka, Japan

**Keywords:** *Clostridium perfringens*, Sepsis, Left hepatic trisectionectomy, Liver abscess, Hemolysis, Perihilar cholangiocarcinoma

## Abstract

**Background:**

*Clostridium perfringens* sepsis has been reported to have a rapid onset and severe clinical outcome. We herein report a case of *C. perfringens* sepsis associated with massive intravascular hemolysis after left hepatic trisectionectomy for perihilar cholangiocarcinoma.

**Case presentation:**

A 72-year-old woman underwent left hepatic trisectionectomy for perihilar cholangiocarcinoma. Her postoperative course was uneventful except for bile leakage. She was discharged on postoperative day (POD) 35. On POD 54, she was readmitted because of abdominal pain with a high fever. Although her vital signs were stable on arrival at the hospital, a laboratory examination showed a severe inflammatory reaction and hemolysis, and she had developed disseminated intravascular coagulation. Abdominal contrast-enhanced computed tomography showed a 70-mm irregular shape and low-density containing air in liver segment 6, which suggested a liver abscess. The abscess was immediately drained of pus containing air. The pus showed multiple Gram-positive bacilli, and two blood cultures showed Gram-positive bacilli and hemolysis. Empirical antibiotic therapy with vancomycin and meropenem was started because *C. perfringens* was detected from the preoperative bile culture. Four hours after arrival, tachypnea and decreased oxygen saturation were observed. Her general condition deteriorated rapidly with significant hypoglycemia, progressive acidosis, anemia, and thrombocytopenia. Despite rapid drainage and empiric therapy, she died six hours after her arrival. At autopsy, the abscess consisted of coagulation necrosis of liver cells with inflammatory cell infiltration, and clusters of Gram-positive large bacilli were observed in the necrotic debris. *C. perfringens* was detected in the drainage fluid and blood culture. She was diagnosed with a liver abscess and severe sepsis caused by *C. perfringens* and treated promptly, but the disease progressed rapidly and led to her death.

**Conclusions:**

Sepsis caused by *C. perfringens* can progress rapidly and lead to death in a few hours, so prompt treatment is needed. When patients who have undergone highly invasive hepatobiliary-pancreatic surgery show hemolysis and hepatic abscesses with gas, *C. perfringens* should be considered the most likely bacterium.

## Background

*Clostridium perfringens* is a bacterium generally found in the human gastrointestinal tract and genitourinary tract in healthy patients [1]. However, *C. perfringens* sepsis has been reported to have a rapidly worsening course and be potentially fatal in patients with immunosuppression [2]. We herein report a patient who developed *C. perfringens* sepsis associated with massive intravascular hemolysis after left hepatic trisectionectomy for perihilar cholangiocarcinoma.

## Case presentation

**Fig. 1:**
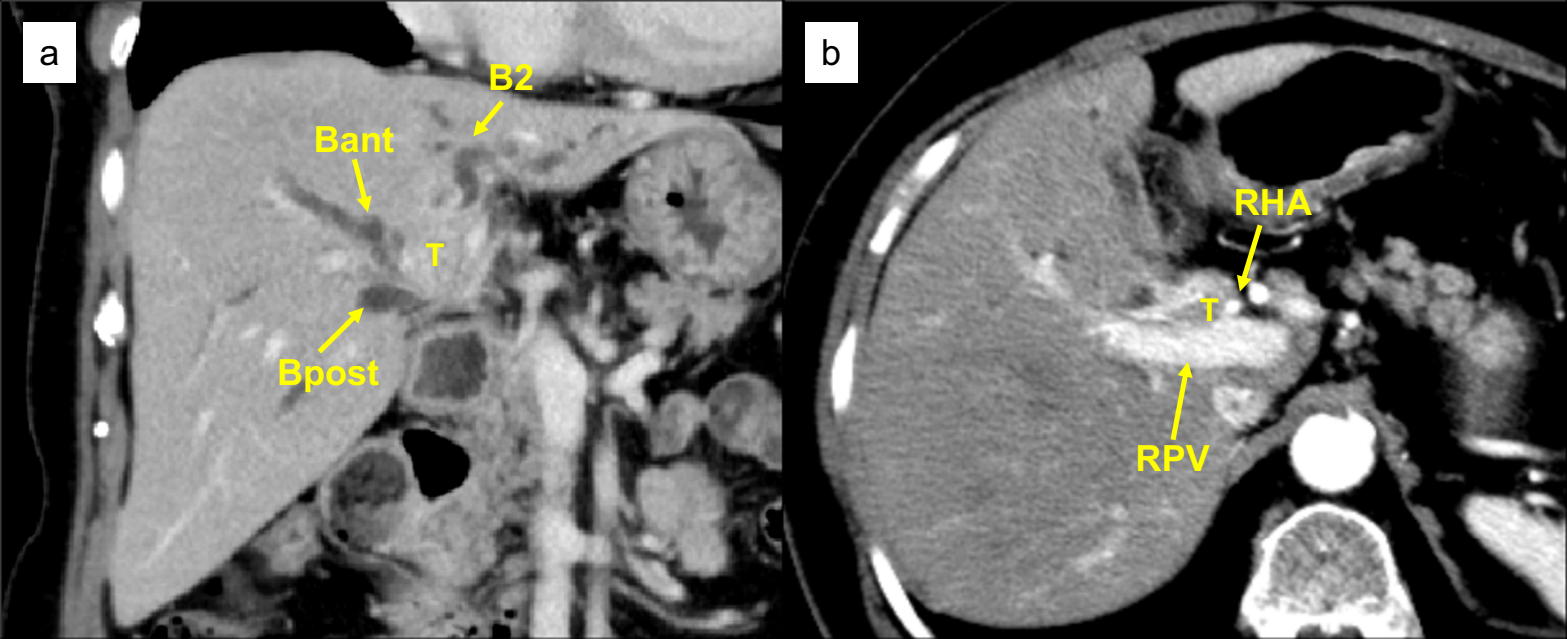
Preoperative contrast-enhanced computed tomography findings. The tumor (T) was mainly located in the left hepatic duct (**a**) and involved the right hepatic artery and the portal vein (**b**). *T* tumor, *B2* segment 2 branch of the bile duct, *Bant* anterior branch of the bile duct, *Bpost* posterior branch of the bile duct, *RHA* right hepatic artery, *RPV* right portal vein

**Fig. 2:**
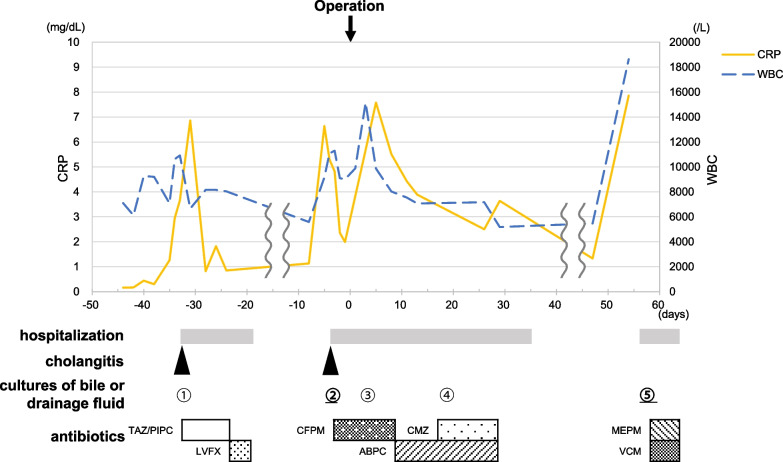
Clinical course. The blue dotted line represents the white blood cell (WBC) count (right y-axis), and the yellow line represents the C-reactive protein (CRP) level (left y-axis). The patient required hospitalization and antibiotic therapy to treat preoperative cholangitis, postoperative bile leakage, and postoperative liver abscess. The numbers of “cultures of bile or drainage fluid” correspond to Table 1. *TAZ/PIPC* tazobactam/piperacillin, *LVFX* levofloxacin, *CFPM* cefepime, *ABPC* ampicillin, *CMZ* cefmetazole, *MEPM* meropenem, *VCM* vancomycin

**Fig. 3:**
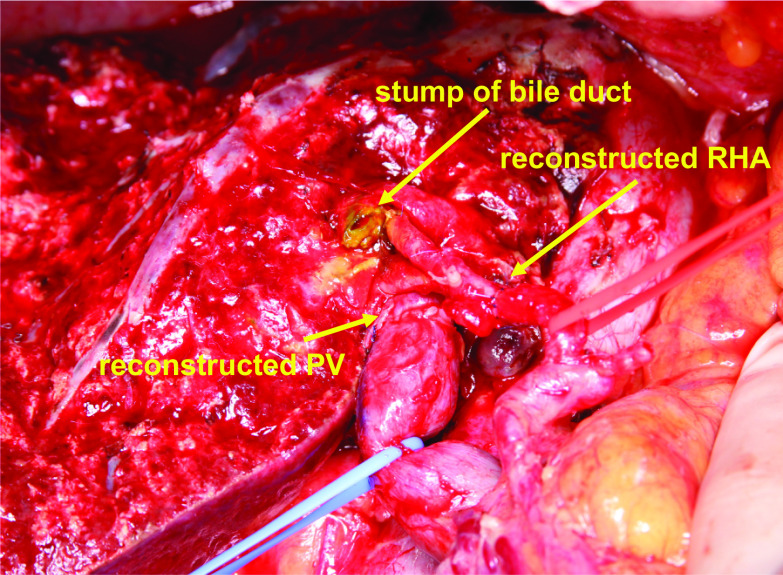
Intraoperative picture after removal of the specimen. *PV* portal vein, *RHA* right hepatic artery

A 72-year-old woman was referred to our hospital for treatment of perihilar cholangiocarcinoma. She had no comorbidities other than hypertension and hyperlipidemia. Multidetector computed tomography (MDCT) demonstrated a 2 cm mass around the left hepatic duct with extensive vascular invasion. The lateral extension reached the confluence of the anterior and posterior branches of the bile duct, representing a Bismuth type IV tumor. The right hepatic artery was involved. The portal vein bifurcation was also involved with obstruction of the left portal vein (Fig. 1). She underwent endoscopic nasobiliary drainage (ENBD) to relieve jaundice, but subsequently developed cholangitis and was treated with antibiotic therapy based on the results of each culture (Fig. 2). The ENBD tube remained in place until the day of surgery (37 days). Cultures of bile from the ENBD tube obtained at each instance of cholangitis revealed *Klebsiella oxytoca*, *Escherichia coli*, *Enterobacter cloacae*, *Enterococcus faecium*, *Enterococcus casseliflavus*, *Bacteroides fragilis*, and *C. perfringens* (Table 1). Given the tumor extension, patient’s general condition (performance status of 0), nutritional status (albumin, 3.8 g/dl), and liver function (indocyanine green clearance, 0.210 and 15-min retention rate, 4.3%), left hepatic trisectionectomy and caudate lobectomy with simultaneous resection of the portal vein and right hepatic artery were planned. Portal vein embolization was performed, and the subsequent remnant liver volume increased from 434 ml (41.9%) to 509 ml (47.5%). Five days prior to surgery, a decreased bile outflow from the ENBD tube and subsequent high fever were observed. We considered postponing the surgery, but since the fever was temporary, the decrease in the bile outflow was caused by the bending of the ENBD tube, and the outflow improved when the bending was released, and the blood tests on the day before surgery were generally within the acceptable range (WBC, 8010 μl; CRP: 1.99 mg/dl), we decided to proceed with the surgery as scheduled. The most recent preoperative bile cultures showed only *Enterobacter cloacae*. Therefore, CFPM was administered prophylactically, targeting this organism according to the infectious disease physician’s instructions. Surgery was performed with an operating time of 10 h 28 min and a blood loss of 2153 ml. (Fig. 3). Her postoperative course was almost uneventful except for bile leakage with International Study Group of Liver Surgery grade B. *C. perfringens* was not detected by ascites and bile culture after surgery. Findings from blood tests 2 days before discharge were generally acceptable (WBC, 7640/μl; CRP, 2.65 mg/dl; albumin, 2.6 g/dl). She was discharged on postoperative day (POD) 35 (Fig. 2). The tumor was pT4 N0 M0 pStage IIIB according to the UICC 8th guidelines [3].

**Table 1:** Microorganisms isolated from perioperative bile or drainage fluid culture

①	②	③	④	⑤
*E. aerogenes* *C. freundii* *K. pneumoniae* *E. faecium* *E. casseliflavus* *E. avium* *Candida* species *α-Streptococcus* *Lactococcus garvieae*	*K. oxytoca* *E. coli* *E. cloacae* *E. faecium* *E. casseliflavus* *Bacillus* species ***C. perfringens***	*E. faecium* *E. casseliflavus* *E. coli* (ESBL+) *K. oxytoca* *B. fragilis* *E. faecalis*	*E. faecium* *E. casseliflavus* *E. coli* (ESBL+)	*E. coli* (ESBL+) ***C. perfringens***

The numbers correspond to Fig. 2. *E. aerogenes; Enterobacter aerogenes, C. freundii; Citrobacter freundii, K. pneumoniae; Klebsiella pneumoniae, E. faecium; Enterococcus faecium, E. casseliflavus; Enterococcus casseliflavus, E. avium; Enterococcus avium, K. oxytoca; Klebsiella oxytoca, E. coli; Escherichia coli, E. cloacae; Enterobacter cloacae, B. fragilis; Bacteroides fragilis, E. faecalis; Enterococcus faecalis*, ESBL; extended-spectrum β-lactamase

**Fig. 4:**
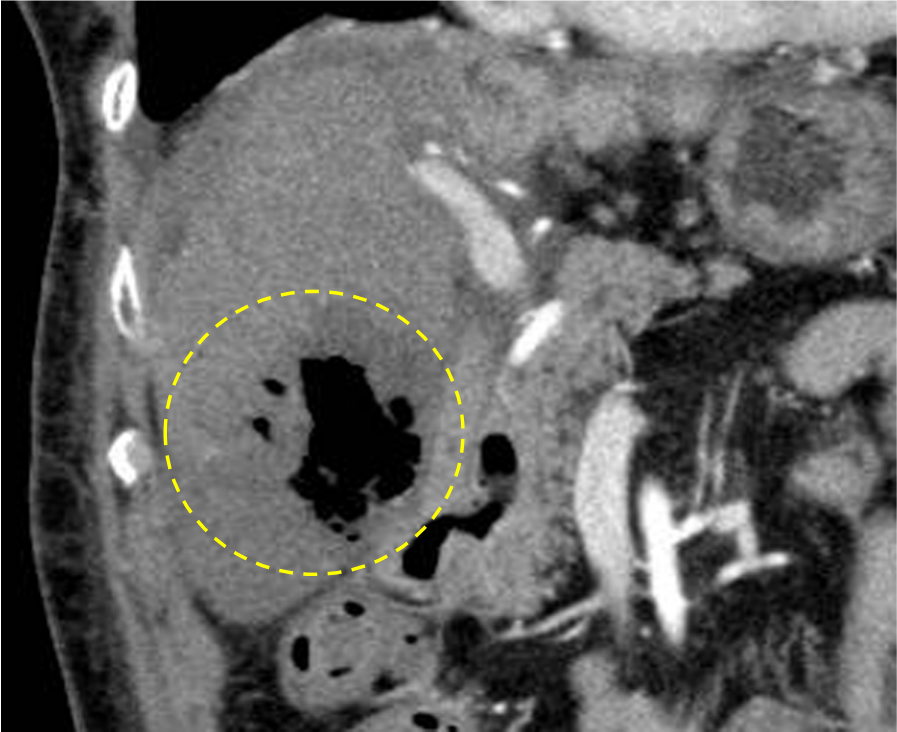
Contrast-enhanced computed tomography findings on POD 54. A liver abscess 70 mm in size was found in segment 6. There was no intrahepatic bile duct dilatation or ischemic areas in the remnant liver

Nineteen days after discharge (POD 54), she was readmitted due to acute-onset abdominal pain, massive hematuria, and high fever from the previous day. On arrival at the hospital, although her vital signs were stable, a laboratory examination showed a severe inflammatory reaction and hemolysis, which suggested disseminated intravascular coagulation (DIC) with an International Society on Thrombosis and Hemostasis DIC score of 7 (Table 2). Abdominal contrast-enhanced CT showed a liver abscess 70 mm in size in segment 6 (Fig. 4), and ultrasonically guided drainage was performed immediately. The abscess was drained of 250 mL of air-filled pus. Multiple Gram-positive bacilli were detected in the pus. Two blood cultures showed Gram-positive bacilli and hemolysis. Based on the strong hemolytic findings, empiric antibiotic therapy (vancomycin: 750 mg/day, meropenem: 3 g/day) was started, targeting *C. perfringens* and β-hemolytic streptococci.

**Table 2:** Laboratory result at readmission

WBC	18,640	/μg
Hb	7.4	g/dL
Plt	6.7	× 10^4^/μL
CRP	7.86	mg/dL
AST	864	U/L
ALT	276	U/L
T-Bil	10.6	mg/dL
BUN	24.0	mg/dL
Cre	0.76	mg/dL
PT	21.9	sec
PT-INR	1.83	
APTT	58.3	s
FDP	465.1	μg/mL
Fibrinogen	237	mg/dL
pH	7.427	
pCO2	28.0	mmHg
pO2	88.4	mmHg
BASE	− 5.5	mmol/L
HCO3-	18.1	mmol/L
Lac	7.2	mmol/L
Glu	66	mg/dL

Four hours after her arrival, however, tachypnea and decreased oxygen saturation appeared. A laboratory examination showed significant hypoglycemia, progressive acidosis, anemia, and thrombocytopenia. Five hours after her arrival, she presented with sudden cardiopulmonary arrest, and cardiopulmonary resuscitation (CPR) was started. Despite CPR being performed for one hour, she died six hours after her arrival.

**Fig. 5:**
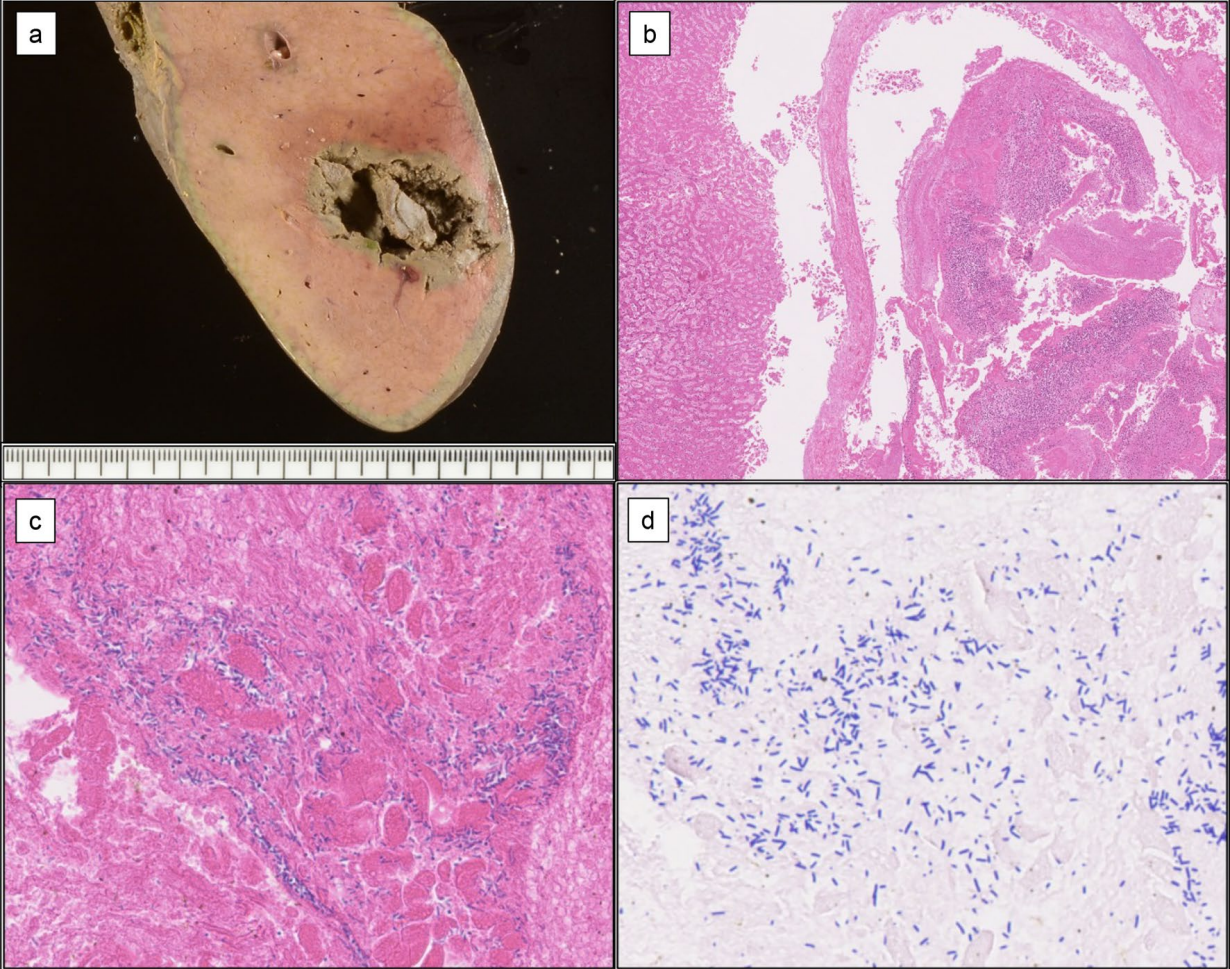
**a** Macroscopic findings of the autopsy liver. A large abscess 32 × 22 mm in size is seen in liver segment 6. **b**–**d** Microscopic findings of the liver abscess. The abscess is composed of coagulation necrosis of liver cells at the periphery and a central cavity with necrotic debris containing many basophilic, Gram-positive large bacilli

At autopsy, an abscess 32 × 22 mm in size was observed in liver segment 6 (Fig. 5a). Microscopically, the abscess consisted of coagulation necrosis of liver cells with acute inflammatory cell infiltration and a cavity (Fig. 5b). Clusters of basophilic, Gram-positive large bacilli were observed in the necrotic debris in the cavity (Fig. 5c, d). *C. perfringens* was detected in the drainage fluid. No ischemic changes due to reconstructed vessel occlusion were observed in the remnant liver. There were no findings of anastomotic leakage at the hepaticojejunostomy. No abscesses were found in any other organs. In summary, the autopsy, along with the clinical, laboratory, and microbiological data, revealed serious *C. perfringens* infection characterized by liver abscesses, sepsis, and DIC.

## Discussion

*C. perfringens* is reported to cause cholecystitis, liver abscess, intrauterine infection, and pyelonephritis. Alpha toxin, which is the most important exotoxin produced by *C. perfringens*, can lyse host cell membranes [4]. This reaction causes many pathological processes, such as tissue necrosis, localized edema, electrolyte disturbance, DIC, severe hemolysis, and multiple organ failure [5]. *C. perfringens* sepsis is thus considered to have a poor prognosis, often being associated with shock and death. The mortality rate of patients with sepsis caused by *C. perfringens* has been reported to be approximately 70–80%, with a 30-day mortality rate of 27–44%. The median time to death from *C. perfringens* is approximately 9.7 h [6]. Indeed, in our patient, despite prompt drainage being performed, sepsis and hemolysis had already developed at the time of arrival, and the duration from arrival to death was only approximately 6 h.

*C. perfringens* is isolated from bile in 4.3% of severe bile duct infections [7]. Sepsis caused by *C. perfringens* rarely occurs in healthy individuals and is more likely to occur in patients with some risk factors, such as advanced age, poorly controlled diabetes, liver cirrhosis, malignancies, surgery, and immunosuppression [8–10]. In the present case, in addition to highly invasive surgery, perioperative complications requiring prolonged perioperative antibiotic therapy for preoperative cholangitis and postoperative bile leakage were assumed to be associated with the development of sepsis due to *C. perfringens*. Prolonged perioperative antibiotic therapy leads to the emergence of drug-resistant bacteria and dysbiosis, which directly impact the immune response balance [11]. Therefore, it was necessary to recognize that the patient remained a so-called “compromised host” and to urge her to be alert for physical changes, even after the early postoperative complications recovered. In addition, in retrospect, CT should have been performed one month before discharge, as some signs of liver abscess might have been detected if we had done so.

Liver abscess can occur via the bile ducts or vessels (arterial or portal) or directly. In our case, there were no clinical findings suggesting enteritis. Neither CT nor autopsy findings indicated enteritis, residual abscess, hepatic ischemia and infarction, or stricture of the hepatojejunal anastomosis. Therefore, bile stasis due to retrograde cholangitis was considered the main cause of subsequent liver abscess and sepsis. Retrograde cholangitis is one of the most common complications in patients after surgery with bile duct reconstruction [12–14]. It is common practice to reference previous culture findings when selecting antibiotic agents [12, 13]. However, while several culture studies were performed, *C. perfringens* was detected only once before surgery and was not found in the latest culture. Prolonged antibiotic therapy may have contributed to the difficulty in detecting *C. perfringens*.

Between 2000 and 2022, 16 sepsis cases were reported in PubMed with the keywords “*Clostridium perfringens*”, “sepsis”, and “postoperative”. Table 3 shows the reported cases of sepsis caused by *C. perfringens* after hepatobiliary-pancreatic surgery [15–29], including our case. The median age was 66 (range 52–82) years old. Nine patients (50%) had comorbid diseases, including 4 (44%) with diabetes mellitus and 11 (69%) with malignant diseases. The operative procedure included pancreatectomy, liver resection, liver transplantation, and cholecystectomy, and 8 patients received bile duct reconstruction. The timing of the onset after surgery has varied among cases. Eight cases (50%) were diagnosed more than 1 month after surgery, while 1 occurred more than 20 years later. Seven cases (44%) were fatal, and 4 (25%) died within 6 h of the diagnosis. Nine cases (53%) required drainage. Whether *C. perfringens* has been detected previously in these reported cases is unclear. However, even in the absence of previous detection, when hemolysis or abscesses with gas are found, treatment should be initiated promptly, keeping *C. perfringens* in mind.

**Table 3:** Reported cases of sepsis caused by C. perfringens after hepatobiliary-pancreatic surgery

Case	Year	Author	Age/sex	Comorbidities	Primary disease	Operative procedure	Onset	Hemolysis	Abscess	Drainage	Outcome	Time to death^*^
1	2006	Leeda [15]	59/M	–	PC	Hepaticojejunostomy and duodenojejunostomy	–	No	Unknown	No	Death	40 h
2	2007	Lochman [16]	–	–	Cholecystolithiasis	Cholecystectomy	48 h	No	Abdominal wall	–	–	
3	2009	Tabarelli [17]	65/F	–	PC	PD	A few days	No	Liver	No	Death	27 days
4	2009	Jandík [18]	–	–	NA	Cholecystectomy	48 h	No	Abdominal wall	Yes	Alive	
5	2009	Daiz [19]	52/M	Hepatitis C	HCC	LT	4 mo	No	None	No	Alive	
6	2011	Juntermanns [20]	55/M	DM	LC	LT	8 YR	No	Skin and soft tissue	Yes	Death	6 h
7	2012	Watt [21]	52/M	–	NA	LT	–	Yes	Unknown	-	Alive	
8	2012	Qandeel [22]	59/M	DM	Gallstone	Cholecystectomy	7 days	No	Liver	Yes	Alive	
9	2014	Kitterer [23]	71/M	–	LC	LT	20 YR	No	Liver	Yes	Death	13 h
10	2018	Martí [24]	66/M	–	Uncinate process adenocarcinoma	PD	2 mo	Yes	Liver	No	Death	3 h
11	2018	Martí [24]	63/M	–	BDC	PD	9 YR	Yes	Liver	No	Death	6 h
12	2018	Ono [25]	82/M	DM, HT, AF, angina pectoris	HCCA	Hepatectomy and bile duct resection	3 mo	No	Liver	Yes	Alive	
13	2020	Hamura [26]	69/M	Hepatitis B	HCC, cholecystitis	Hepatectomy	15 days	No	Subphrenic space	Yes	Alive	
14	2020	Dahl [27]	68/M	HT	PC	TP	1 mo	No	Liver	Yes	Alive	
15	2022	Takahashi [28]	70/M	DM	Ampulla of Vater carcinoma	PD	16 days	Yes	Liver	Yes	Alive	
16	2022	Itoh [29]	77/M	HT	HCCA	Hepatectomy and bile duct resection	1 YR	No	Recurrent tumor at the liver	No	Alive	
Our case			72/F	HT	HCCA	Hepatectomy and bile duct reconstruction	54 days	Yes	Liver	Yes	Death	6 h

*PC* pancreatic cancer, *HCC* hepatocellular carcinoma, *LC* liver cirrhosis, *BDC* distal bile duct carcinoma, *HCCA* hilar cholangiocarcinoma, *PD* pancreaticoduodenectomy, *LT* liver transplantation, *TP* total pancreatectomy, *DM* diabetes mellitus, *HT* hypertension, *AF* atrial fibrillation

^*^The duration from arrival to death

## Conclusions

Sepsis caused by *C. perfringens* can progress rapidly and lead to death in a few hours. When patients develop hemolysis and air-filled liver abscesses after highly invasive hepatobiliary-pancreatic surgery, especially combined bile duct reconstruction, *C. perfringens* should be considered a potential cause and should receive prompt multidisciplinary treatment.

## Data Availability

All data analyzed in this study are included in this manuscript.

## Abbreviations

MDCT: Multidetector computed tomography
ENBD: Endoscopic nasobiliary drainage
UICC 8th: The Union for International Cancer Control classification 8th edition
POD: Postoperative day
DIC: Disseminated intravascular coagulation
CPR: Cardiopulmonary resuscitation
